# IgM Repertoire Biodiversity is Reduced in HIV-1 Infection and Systemic Lupus Erythematosus

**DOI:** 10.3389/fimmu.2013.00373

**Published:** 2013-11-15

**Authors:** Li Yin, Wei Hou, Li Liu, Yunpeng Cai, Mark Andrew Wallet, Brent Paul Gardner, Kaifen Chang, Amanda Catherine Lowe, Carina Adriana Rodriguez, Panida Sriaroon, William George Farmerie, John William Sleasman, Maureen Michels Goodenow

**Affiliations:** ^1^Department of Pathology, Immunology and Laboratory Medicine, College of Medicine, University of Florida, Gainesville, FL, USA; ^2^Florida Center for AIDS Research, University of Florida, Gainesville, FL, USA; ^3^Department of Biostatistics, College of Public Health, University of Florida, Gainesville, FL, USA; ^4^Interdisciplinary Center for Biotechnology Research, University of Florida, Gainesville, FL, USA; ^5^Division of Infectious Diseases, College of Medicine, University of South Florida, St. Petersburg, FL, USA; ^6^Department of Pediatrics, Division of Allergy, Immunology, and Rheumatology, College of Medicine, University of South Florida, St. Petersburg, FL, USA

**Keywords:** IgM antibody transcriptome repertoire, biodiversity, HIV-1, systemic lupus erythematosus, somatic hypermutation, naïve B cells, IgM memory B cells, pyrosequencing

## Abstract

**Background**: HIV-1 infection or systemic lupus erythematosus (SLE) disrupt B cell homeostasis, reduce memory B cells, and impair function of IgG and IgM antibodies.

**Objective**: To determine how disturbances in B cell populations producing polyclonal antibodies relate to the IgM repertoire, the IgM transcriptome in health and disease was explored at the complementarity determining region 3 (CDRH3) sequence level.

**Methods**: 454-deep pyrosequencing in combination with a novel analysis pipeline was applied to define populations of IGHM CDRH3 sequences based on absence or presence of somatic hypermutations (SHM) in peripheral blood B cells.

**Results**: HIV or SLE subjects have reduced biodiversity within their IGHM transcriptome compared to healthy subjects, mainly due to a significant decrease in the number of unique combinations of alleles, although recombination machinery was intact. While major differences between sequences without or with SHM occurred among all groups, IGHD and IGHJ allele use, CDRH3 length distribution, or generation of SHM were similar among study cohorts. Antiretroviral therapy failed to normalize IGHM biodiversity in HIV-infected individuals. All subjects had a low frequency of allelic combinations within the IGHM repertoire similar to known broadly neutralizing HIV-1 antibodies.

**Conclusion**: Polyclonal expansion would decrease overall IgM biodiversity independent of other mechanisms for development of the B cell repertoire. Applying deep sequencing as a strategy to follow development of the IgM repertoire in health and disease provides a novel molecular assessment of multiple points along the B cell differentiation pathway that is highly sensitive for detecting perturbations within the repertoire at the population level.

## Introduction

HIV-1 infection and systemic lupus erythematosus (SLE) each results in defective B cell activation and differentiation (1, 2). Both conditions have decreased proportions of CD27^+^ class switched memory B cells, increased B cell apoptosis, and increased expression of B cell activation markers (3–6). In HIV infection, disruption of B cell homeostasis and loss of normal B cell architecture within lymphoid tissues occur early in disease and persist even with control of viral replication following antiretroviral therapy (ART) (7, 8). Individuals with SLE display defects in B cell checkpoints in both early and late stage development contributing to impaired tolerance and autoantibody production (9).

The dynamics between defects in B cell function and underlying molecular perturbations in the B cell repertoire during the course of infection or autoimmunity have not been extensively evaluated, reflecting in part the challenge of generating sufficiently robust data sets by conventional clonal sequencing. Massively parallel deep sequencing has revolutionized the capacity to evaluate the depth and breadth of the immunoglobulin (Ig) repertoire (10). Application of deep sequencing to probe the Ig heavy chain variable region (IGH) repertoire along the B cell developmental pathway may pin point HIV-mediated defects over the antibody maturation, and uncover evidence for elusive broadly neutralizing HIV-specific antibodies (bn-HIV-Ab) critical to development of an effective HIV vaccine. This study focused on IGHM repertoire at early B cell developmental stage before isotype switch. IgM is the initial antibody generated when encountering antigen. Activated IgM B cells confer substantial response in acute HIV-infection (11–13). IgM antibodies demonstrated broad spectrum and high affinity to HIV-1 envelope glycoproteins, neutralized HIV-1 more potently than IgG due to pentameric binding nature, and prevented viral entry across the mucous membrane (14, 15).

The limited repertoire of bn-HIV-Ab share biochemical, structural, and functional features resembling natural polyreactive autoantibodies and are produced by individuals with autoimmunity, particularly SLE, and a significant proportion of HIV-infected individuals (16–25). Polyreactive autoantibodies and bn-HIV-Ab are frequently IgG (26–28), or can be IgA (29–32). More than a third of SLE individuals have IgM antibodies reactive with HIV-1 gp41-derived peptides (33), a CD4-reactive IgM Fab clone isolated from a healthy individual inhibited HIV-1 replication (34), and IgM autoantibodies blocked HIV-1 entry (35).

While polyreactive antibodies are well-characterized at the biochemical level, molecular assessment of the Ig repertoire among populations of B cells that produce polyreactive antibodies is limited. Our current in-depth analysis based on deep sequencing of the IgM transcriptome was designed to examine the molecular repertoire of IGH complementarity determining region 3 (CDRH3) in IgM among individuals with SLE or HIV-1 infection and relate the findings to a group of healthy individuals by analysis of biodiversity. Biodiversity is used in population genetics to present a unified view of variation of life forms within habitats based on the premise that genomes are taxonomically similar, randomly distributed, and sufficiently large (36, 37). Assessments of biodiversity from deep sequencing data provide unprecedented views of the richness of immune loci in primates, zebra fish, and humans (38–41). The goal for our study was to determine how disturbances in B cell populations producing polyclonal antibodies relate to biodiversity of the IgM repertoire by examining key components, including allele usage, V-D-J recombination and junctional diversity, and extent of somatic hypermutation (SHM), which collectively contribute to differences in Ig biodiversity between health and disease.

## Materials and Methods

### Study cohort

Sixteen individuals, enrolled in a protocol approved by the Institutional Review Boards of the University of Florida and the University of South Florida, included four groups (*n* = 4 per group): healthy controls (HC), subjects with SLE, and HIV-1 infected individuals either therapy-naïve (HIV) or receiving combination antiretroviral therapy (cART) (HIVTx) (Table 1). Groups within the cohort were age-balanced with median (25–75% quartile range) age of 20 (16–22) years. SLE subjects were untreated and diagnosed by clinical and laboratory criteria defined by the American College of Rheumatology (42). Therapy-naïve subjects were HIV-1 infected by sexual transmission for at least 6 months (Table 1). HIVTx individuals were infected either through maternal to child transmission or by contaminated blood products for 16.0 (12.3–21.3) years, treated for 8.0 (1.5–17.5) years, and achieved viral suppression (log_10_ HIV-1 RNA copies/ml < 1.7), and CD4 > 25% for 1.9 (0.5–6.0) years at the time of study (Table 1). No subject received any vaccination 30 days prior to study entry, and/or had acute infection to reduce the chance of plasma cell circulating in peripheral blood. Informed consent was obtained from all subjects.

**Table 1:** **Demographic characterization of study cohort**.

Study group	Sex		Age (year)	Length of infection (year)	ART length (year)	CD4 %	Viral load^a^	Length of viral suppression (year)	B cell count (cells/μl)	CD27^−^IgM B cells (%)	CD27^+^ IgM B cells (%)
		
	M	F	Median (25–75% quartile range)								
HC	2	2	22 (18–22)	N/A	N/A	N/A	N/A	N/A	49 (37–112)	68 (52–83)	33 (17–48)
SLE	1	3	15 (14–20)	N/A	N/A	N/A	N/A	N/A	72 (18–165)	78 (75–85)	22 (15–25)
HIV	1	3	22 (17–24)	0.8 (0.5–2.5)	Naive	34 (26–53)	4.0 (2.5–4.9)	N/A	254 (124–308)	56 (52–81)	44 (19–48)
HIVTx	1	3	20 (15–25)	16.0 (12.3–21.3)	8.0 (1.5–17.5)	29 (24–31)	1.7 (1.7–1.7)	1.9 (0.5–6.0)	93 (57–440)	63 (60–71)	37 (29–40)

*^a^ Log_10_ HIV-1 RNA copies/ml plasma; N/A, not applicable*.

*HC, healthy control group; SLE, subjects with systemic lupus erythematosus; HIV, therapy-naïve HIV-infected subjects; HIVTx, HIV-infected individuals with antiretroviral treatment*.

### B cell profiles by multiparameter flow cytometry

Immunofluorescence staining was performed using the whole blood lysis method (43) and analysis by LSR2 flow cytometer (BD Biosciences, Franklin Lakes, NJ, USA) with Diva software (BD Biosciences, San Jose, CA, USA). Monoclonal antibody panel included pan B cell marker PE Cy7-conjugated anti-CD19 (BD Biosciences), memory B cell marker Qdot 655-conjugated anti-CD27 (Invitrogen, Carlsbad, CA, USA), and surface IgM with APC-conjugated anti-IgM (BD Biosciences). B cell percentages ranged from 6.6 to 16.1% of peripheral blood mononuclear cells (PBMC) across the groups, CD19^+^ IgM B cells ranged from 70 to 90% of total B cells (Table 1).

### Generation of IGHM CDRH3 amplicon libraries

**Figure 1:**
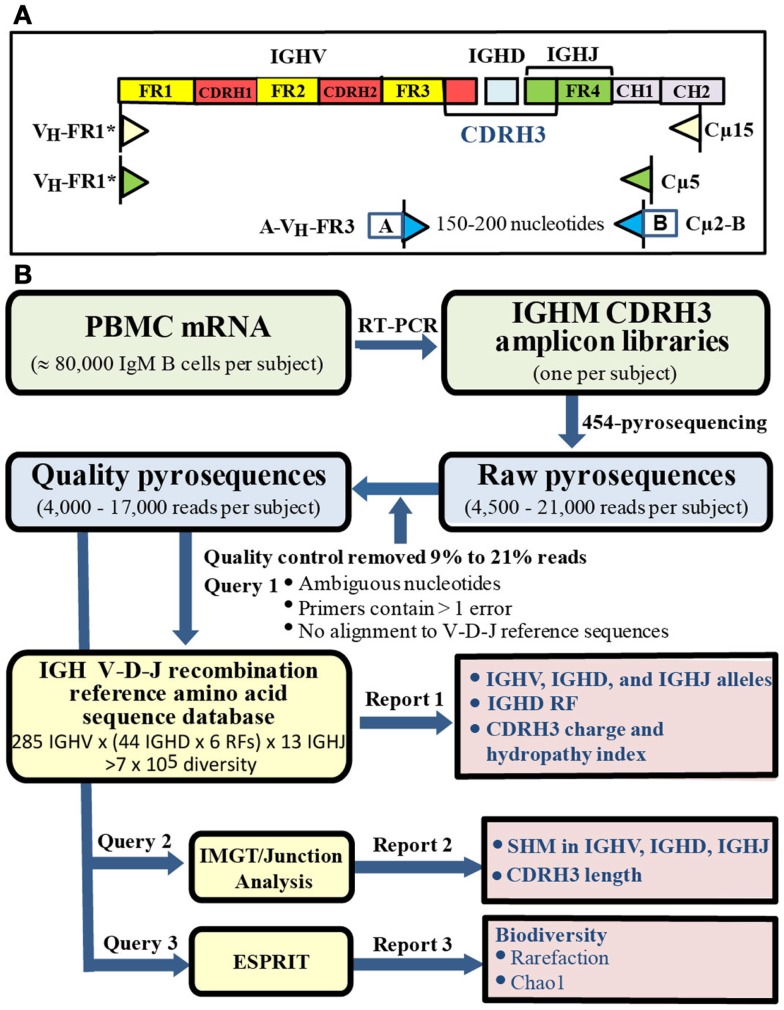
**Amplicon libraries and bioinformatic pipeline**. **(A)** Library construction. IgM specificity was determined by reverse primers sequences specific for IgM constant region without IgM B cell purification. **(B)** Bioinformatic pipeline. Quality sequences (blue box) were analyzed by querying: (1) customized IGH V-D-J recombination reference sequence database, (2) IMGT/Junction Analysis, (3) ESPRIT (yellow boxes) to generate three reports (pink boxes).

Analysis of the IGHM CDRH3 transcriptome was performed using total peripheral blood mRNA without isolation of IgM B cells. Specificity for the IgM transcriptome was achieved by reverse amplification primers Cμ15, Cμ5, and Cμ2 homologous to the IgM constant region (44) (Figure 1A). An amplicon library was constructed for each subject by RT-PCR using SuperScript™ One-Step RT-PCR with Platinum *Taq* (Invitrogen) and GoTaq colorless Master Mix (Promega, Madison, WI, USA) from ∼200 ng mRNA (equivalent to about 100,000 B cells or 80,000 IgM B cells) extracted from total PBMC by MicroPoly[A]Purist™ kit (Ambion, Austin, TX, USA).

Forward inner primer pan V_H_-FR3 is located in framework 3 and does not overlap CDRH3 (44). Using the software Oligo (Molecular Biology Insights, Inc., Cascade, CO, USA), pan V_H_-FR3 primer was predicted to bind with similar capacity to a unique sequence in each IGHV family when evaluated against IGHV family specific reference sequences. In contrast, the pan V_H_-FR1 primer (44) displayed different binding capacity to different IGHV families. Specifically, pan V_H_-FR1 primer bound preferentially to IGHV3; displayed low binding capacity with IGHV4, IGHV5, and IGHV7; failed to bind to IGHV2 or IGHV6; and bound to false priming sites in IGHV1, IGHV4, and IGHV5. To overcome the different binding capacity of the published primer sequence, we designed a modified panV_H_-FR1* primer (CAGGTGCAGCTGG**A**G**C**AGTCTGG_) that was one nucleotide shorter and substituted A and C for T and G in the published primer sequence, respectively. When evaluated by Oligo, the modified panV_H_-FR1* primer displayed similar binding capacity to each IGHV family reference sequence and no binding to false priming sites. Forward and reverse primers, pan V_H_-FR3, and Cμ2, in the final amplification were conjugated at 5′ ends with A (GCCTCCCTCGCGCCATCAG) or B (GCCTTGCCAGCCCGCTCAG) adaptor, respectively, to generate amplicons ranging from 150 to 200 nucleotides. Gel-purified amplicons were submitted to the Interdisciplinary Center for Biotechnology Research (University of Florida) for 454-pyrosequencing using a Genome Sequencer FLX (454 Life Sciences) according to the manufacturer’s protocol.

### Bioinformatics pipeline

A bioinformatics pipeline was developed to facilitate analysis of large numbers of relatively short IGHM CDRH3 sequences that could not be processed by conventional IMGT/V-QUEST analysis (Figure 1B) (45). Raw reads ranged from 4,500 to 21,000 pyrosequences per subject. A quality control step filtered 9–21% low quality reads with ambiguous nucleotides, more than one error in either primer tag, or failure to align to reference sequences in the germ line IGH V-D-J recombination amino acid reference sequence database (see below), leaving 4,000–17,000 quality sequences per sample with no significant difference in number of sequences among the groups (Table 2).

**Table 2:** **Sequence profiles**.

Group	Raw sequences	Removal (%)	Quality sequences	Sequences with 1 nucleotide substitution (%)	Final quality sequences
	
	Mean ± SD				
HC	15,005 ± 2,514	19.9 ± 4.3	11,951 ± 1,490	12.5 ± 0.9	10,460 ± 1,351
SLE	9,613 ± 3,102	16.0 ± 4.7	8,171 ± 2,936	16.3 ± 2.1	6,822 ± 2,411
HIV	7,727 ± 2,236	17.0 ± 5.4	6,341 ± 1,573	13.4 ± 0.9	5,482 ± 1,326
HIVTx	14,746 ± 7,101	18.0 ± 4.4	12,140 ± 5,797	14.8 ± 1.3	10,388 ± 5,034

*HC, healthy control group; SLE, subjects with systemic lupus erythematosus; HIV, therapy-naïve HIV-infected subjects; HIVTx, HIV-infected individuals with antiretroviral treatment*.

To overcome the limitation of IMGT/V-QUEST for high throughput classification of relatively short sequences, a novel custom reference sequence database containing over 700,000 germ line IGH V-D-J amino acid (aa) sequences was established by generating *in silico* all possible combinations of germ line IGHV (285 unique aa sequences), IGHD [44 unique nucleotide sequences translated in 6 reading frames (RF) producing 222 aa sequences], and IGHJ (13 unique aa sequences) alleles downloaded from IMGT (45). Each reference sequence was annotated for IGHV, IGHD, and IGHJ allele use, IGHD RF in the header with C104 and W118, the 5′ and 3′ boarders of CDRH3, marked by aligning to germline IGHV and germline IGHJ reference sequences using FASTA (http://fasta.bioch.virginia.edu/fasta_www2/fasta_down.shtml) (46). After removing adapter sequences, query sequences were then aligned to the reference sequences using FASTY. Use of IGHV, IGHD, and IGHJ alleles, and IGHD RF of each query 454-sequence was extrapolated from the header of the best-matched reference sequence, the C104 and W118 positions of each query sequence were identified, the part of the query sequence matched to the C104-W118 region of the reference sequence was extracted from the entire sequence, and CDRH3 charge and hydropathy index calculated using an in-house code (47, 48). IMGT/Junction Analysis was then performed allowing 3D-GENEs (49). Using this strategy, the frequency distribution of IGHD and IGHJ alleles, with IGHD2, IGHD3 and IGHD6, and IGHJ4 as the dominant genes, was similar to previous reports (50, 51). However, due to the relatively short IGHV sequence, we observed some ambiguity in IGHV4 allele assignment; consequently, no comparisons of IGHV alleles without or with SHM were made.

The total number of mutations including silent- and non-silent-mutations in CDRH3 between C104 and W118 (40) was calculated to summarize extent of SHM as: (ΣSHM within CDRH3 in each SHM^+^ sequence ÷ ΣCDRH3 nucleotide length in each SHM^+^ sequence) × 100 nucleotides. The effect of N and P nucleotides to the SHM was similar among the study groups because the number of N and P nucleotides was minimized when the setting for D-GENES was 3, and the frequency of sequences with one D, D-D fusion and D-D-D fusion was similar among study groups (data not shown).

To avoid potential ambiguity in scoring SHM, an average of 14.3 ± 0.6% of reads among groups with a single nucleotide difference from aligned reference sequences was removed from analysis (Table 2) (40). IGHM sequences with two or more mutations in CDRH3 were classified as SHM^+^ representing IgM memory B cells, while SHM^−^ sequences represent naïve B cells. After correction of deletion and insertion, the PCR and pyrosequencing-induced rate of misincorporation tested in our control analysis of clones of sequences was 0.18 errors per 200 nucleotides, the longest sequence length, similar to other reports (52–54) and well below the level of SHM identified in the IgM populations.

ESPRIT was applied to study the biodiversity (genetic complexity) of nucleotide sequences as well as V-D-J combinations of the IGHM CDRH3 transcriptome repertoire by clustering the sequences at 0% genetic distance (55). Rarefaction analysis measures increase in biodiversity along the depth of sequencing (number of sequences). The deeper the initial slope is, the higher the biodiversity. Left shift or right shift of the curve indicates an increase or decrease of biodiversity. Chao1 analysis inferred maximum biodiversity within the input templates (55, 56). Biodiversity is influenced by input cell number, the coverage (sequence number/input cell number), and clonality (frequency of clusters with more than 10 repeated sequences). Coverage was ∼10% in each individual to minimize the influence of preferential amplification of replicate templates. Biodiversity was weighted by the absolute number of input IgM B cells (calculated from the absolute lymphocyte count multiplied by the percentage of CD19^+^ IgM B cells) to make the data comparable among study groups. To avoid a potential bias from sequence number, clonality was averaged on the same number of sequences randomly drawn 1,000 times from each sequence pool.

To rule out an influence by differences in sequence numbers among individuals, a bootstrap resampling method was used to generate 1,000 rarefaction curves and Chao1 values from random data sets with the same number of sequences for each individual. Calculated and estimated biodiversity derived from averages of 1,000 rarefaction curves produced similar findings (result not shown).

### Statistical analysis

Comparisons among study groups were performed by ANOVA. Kolmogorov–Smirnov test was used to test Gaussian distribution. Overall difference of distribution of CDRH3 length frequencies, and charge and hydropathy along CDRH3 lengths among study groups were evaluated as described (57). Comparison of frequency of sequences in each gene allele, or each CDRH3 length between SHM^+^ and SHM^−^ sequences was performed by paired *t*-test. Pearson correlation evaluated the relationship between frequency of SHM^+^ sequences and frequency of peripheral CD27^+^ IgM B cells. A primary purpose of this pilot study with relatively small sample size was to detect meaningful trends, thus parametric methods instead of conservative non-parametric methods were performed. *P* values were unadjusted for multiple tests to increase sensitivity to identify differences. Statistical analyses were performed using SAS version 9.1 (SAS Institute, Cary, NC, USA) with *p* < 0.05 (two sided) defined as significant.

## Results

### Somatic hypermutations

**Figure 2:**
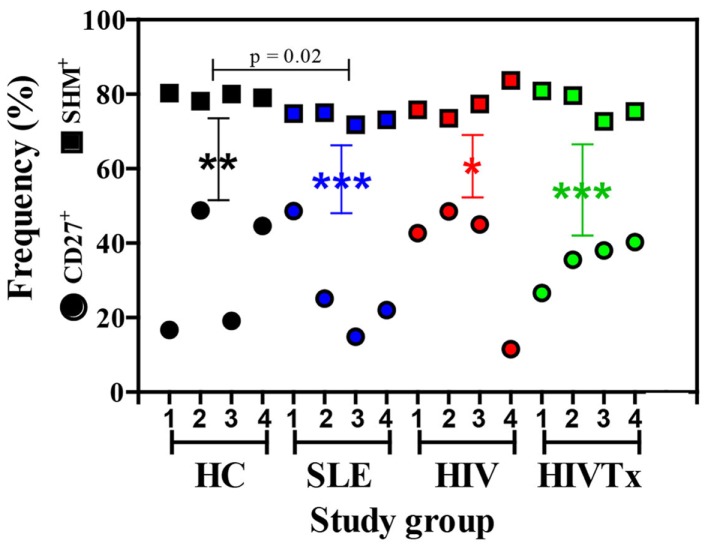
**Frequency of SHM^+^ IgM sequences exceeds the frequency of CD27^+^ IgM B cells in peripheral blood**. Frequency of SHM^+^ IgM sequences in HIV and HIVTx (treated) groups was similar to HC (healthy controls), but significantly reduced in individuals with SLE (systemic lupus erythematosus) (*p* = 0.02). Frequency of SHM^+^ IgM sequences was significantly greater than the proportion of CD27^+^ IgM B cells in each group (HC, **p = 0.001; SLE, ***p < 0.0001; HIV, *p = 0.004; HIVTx, ***p < 0.0001). Reduced frequency of CD27^+^ IgM B cells in SLE individuals compared to HC failed to reach statistical significance. Frequency of SHM^+^ sequence did not correlate with percent of CD27^+^ IgM B cells (*r*^2^ = 0.06, *p* = 0.35). Symbols: square, IGHM CDRH3 sequences with SHM; circle, CD27^+^ IgM B cells.

IGHM CDRH3 sequences were initially classified by absence or presence of SHM as a molecular means to distinguish naïve B cells from IgM memory B cells. Frequency of sequences with SHM in HIV or HIVTx groups was similar to HC group, but significantly reduced in individuals with SLE (*p* = 0.02) (Figure 2). In contrast, extent of SHM (mutations/100 nucleotides) in IGHM CDRH3 among groups was similar. While the proportion of B cells expressing CD27^+^ IgM memory phenotype was similar among groups, percentage of phenotypically mature CD27^+^ IgM B cells was significantly less than the frequency of IgM sequences with SHM within each group (Figure 2). No correlation occurred between the proportion of CD27^+^ B cells and SHM (*r*^2^ = 0.06, *p* = 0.35). Subsequent analysis of the IgM transcriptome was based on molecular assessments of sequences without or with SHM in IGHM CDRH3.

### Biodiversity of IGHM CDRH3 transcriptome repertoire in health and disease

**Figure 3:**
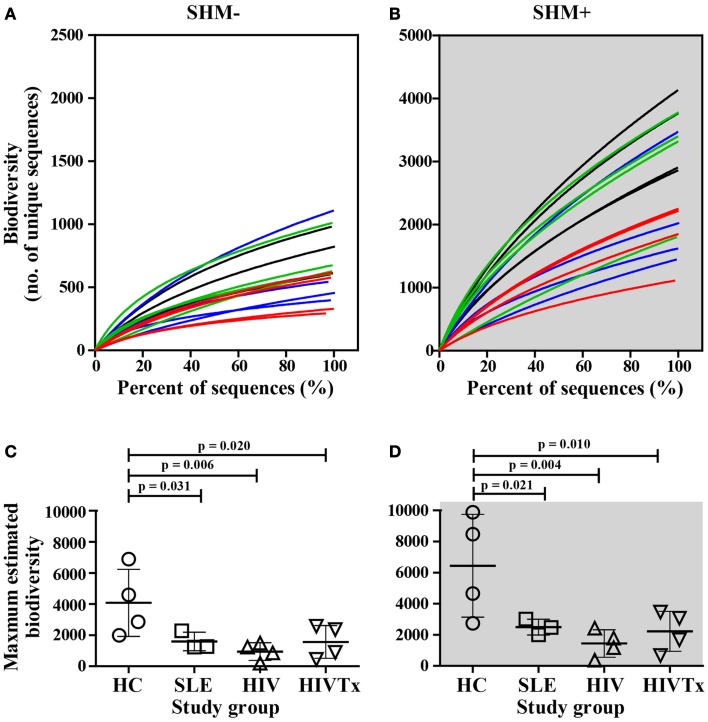
**Greater biodiversity among populations of IGHM CDRH3 sequences in health control group than in groups with SLE or HIV-1 infection in sequences without or with SHM**. Biodiversity in IGHM CDRH3 sequences without **(A)** or with **(B)** SHM in young adults with SLE (blue lines) and among HIV-infected individuals (red lines, untreated; green lines, treated) in comparison to healthy counterparts (black lines). Maximum IGHM CDRH3 biodiversity in input IgM B cells without **(C)** or with **(D)** was significantly lower in SLE (SHM^−^, *p* = 0.031; SHM^+^, *p* = 0.021), HIV (SHM^−^, *p* = 0.006; SHM^+^, *p* = 0.004), and HIVTx (SHM^−^, *p* = 0.020; SHM^+^, *p* = 0.010) in comparison with HC. ART failed to normalize IGHM CDRH3 biodiversity in SHM^−^ or SHM^+^ IGHV CDRH3 repertoire in HIV-infected subjects (HIVTx vs. HIV, *p* > 0.05).

Deep sequencing data sets support application of novel rarefaction analysis to Ig biodiversity that cannot be inferred from analysis of limited numbers of sequences. Rarefaction curves provide comparison of biodiversity along with the depth of sequencing among study groups; deeper slopes indicate greater biodiversity. IGHM CDRH3 among healthy young adults displayed a range of biodiversity that overall was greater than biodiversity in SLE or HIV-1 infection (Figures 3A,B). Differences in biodiversity between health and disease were apparent in populations of IgM sequences without SHM, and to a greater extent among sequences with SHM due to contribution of SHM. At the depth of sequencing, none of the rarefaction curves reached saturation, indicating a preponderance of unique sequences and an extent of IGHM CDRH3 diversity that exceeded the depth of sequencing.

When maximum IGHM CDRH3 biodiversity was inferred, the SHM-negative repertoire within healthy individuals displayed significantly greater maximum biodiversity than SLE (*p* = 0.031), HIV (*p* = 0.006), or HIVTx (*p* = 0.020) (Figure 3C). Likewise, the repertoire with SHM among healthy individuals also displayed significantly greater biodiversity than SLE (*p* = 0.021), HIV (*p* = 0.004), or HIVTx (*p* = 0.010) (Figure 3D). ART failed to normalize biodiversity in IGHM CDRH3 repertoire either without or with SHM in HIV-infected subjects (Figures 3C,D).

Multiple factors contribute to biodiversity. SHM is one factor, but extent of SHM was similar among study groups. Likewise, extent and distribution of clonality were similar among sequences without or with SHM in each study group with a frequency of ∼99% for unique clusters about 0.2% of clusters with more than 10 repeated sequences. Consequently, to identify molecular differences that might contribute to reduced biodiversity without or with SHM among SLE or HIV-infected groups, CDRH3 length variation, charge, and hydropathy distribution, as well as IGHD and IGHJ allelic use, and diversity of allele combinations were investigated.

### CDRH3 length variation

**Figure 4:**
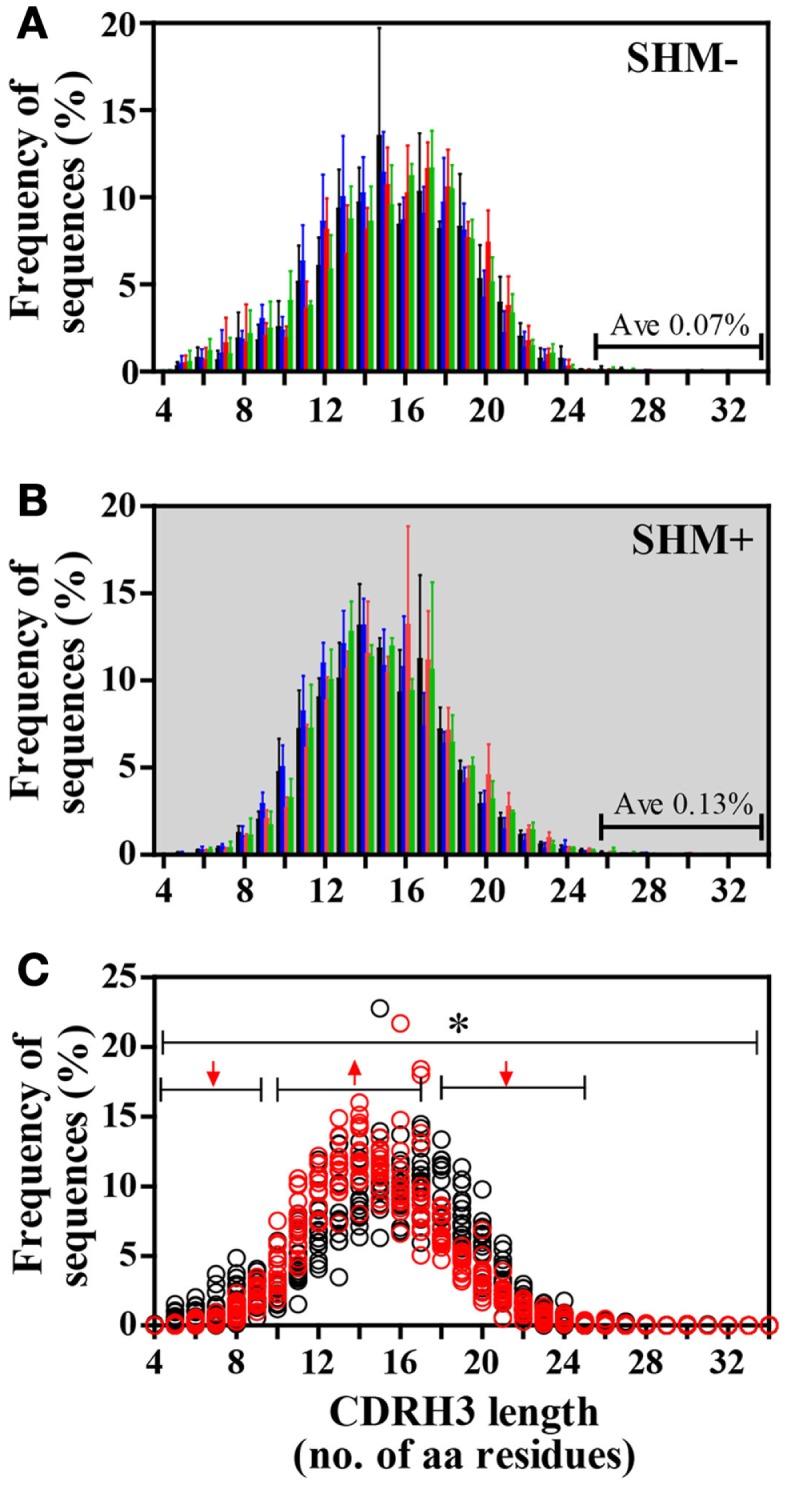
**Frequency distribution of IGHM CDRH3 amino acid lengths differed between IgM B cells without or with SHM**. **(A)** Lengths of SHM^−^ CDRH3 regions displayed a Gaussian distribution ranging from 4 to 31 amino acid residues; lengths of 15 amino acids occurred most frequently. **(B)** Lengths of SHM^+^ CDRH3 regions showed Gaussian distribution ranging from 5 to 34 amino acids with lengths of 14 amino acids used most often. **(C)** Overall length distribution between SHM^−^ and SHM^+^ sequences differed [*p = 0.004]. Among SHM^+^ sequences, shorter lengths [4–9 amino acids] and longer lengths [18–25 amino acids] were expressed significantly less [↓, *p* < 0.0001], while lengths of 10–17 amino acids were used more frequently in comparison to SHM^−^ sequences [↑, *p* < 0.0001]. Bars: HC, black; SLE, blue; HIV-infected individuals: red, untreated; green, treated. Circles: SHM^−^, open black; SHM^+^, open red.

**Figure 5:**
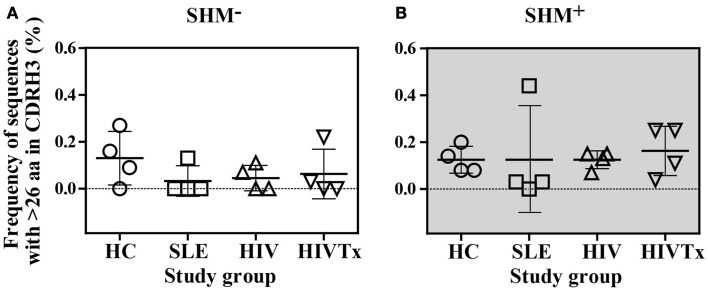
**Similar frequency of sequences with CDRH3 lengths longer than 26 amino acids among study groups**. **(A)** Among SHM^−^ sequences, frequency of sequences with greater than 26 amino acids CDR3 length ranged from 0.03 [± 0.06%] to 0.13% [± 0.12%], which was similar among the four study groups. **(B)**. Among SHM^+^ sequences, frequency of sequences with greater than 26 amino acids CDR3 length ranged from 0.13 [± 0.04%] to 0.16% [± 0.10%], which was similar among the four study groups.

**Figure 6:**
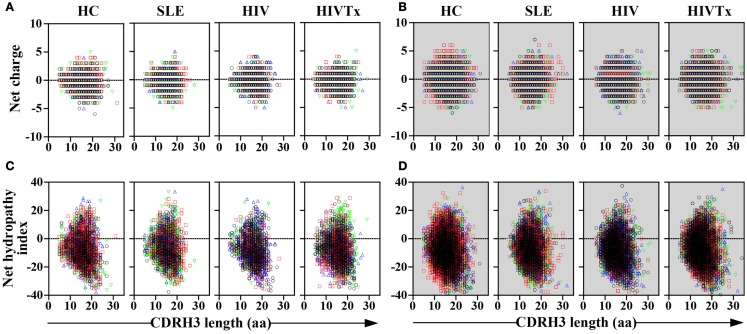
**Similar distribution of charge and hydropathy across IGHM CDRH3 length among study groups**. Distribution of charge and hydropathy across IGHM CDRH3 amino acid length range were similar among the four study groups in sequences without [charge, **(A)**; hydropathy index, **(C)**] or with SHM [charge, **(B)**; hydropathy index, **(D)**].

In all groups, CDRH3 length variation displayed Gaussian distributions (Figure 4). CDRH3 regions without SHM ranged from 4 to 31 amino acid residues with a peak frequency of 15 amino acids (Figure 4A). SHM^+^ CDRH3 regions ranged from 5 to 34 amino acids, with lengths of 14 amino acids occurring most frequently (Figure 4B). Overall length distribution differed significantly between sequences without or with SHM (*p* = 0.004) (Figure 4C). In general, CDRH3 lengths of 10–17 amino acids occurred with greater frequency among sequences with SHM than in sequences without SHM (*p* < 0.0001). The frequency of long CDRH3 regions (27–34 amino acids) ranged from 0.03 (± 0.06%) to 0.13% (± 0.12%) in sequences without SHM and 0.13 (± 0.04%) to 0.16% (± 0.10%) in sequences with SHM with no significant differences among study groups (Figure 5). Charge and hydropathy distribution across CDRH3 amino acid length range were similar among all groups indicating that junctional modifications in sequences without or with SHM were unchanged (Figure 6).

### Allelic frequency of IGHD and IGHJ genes

**Figure 7:**
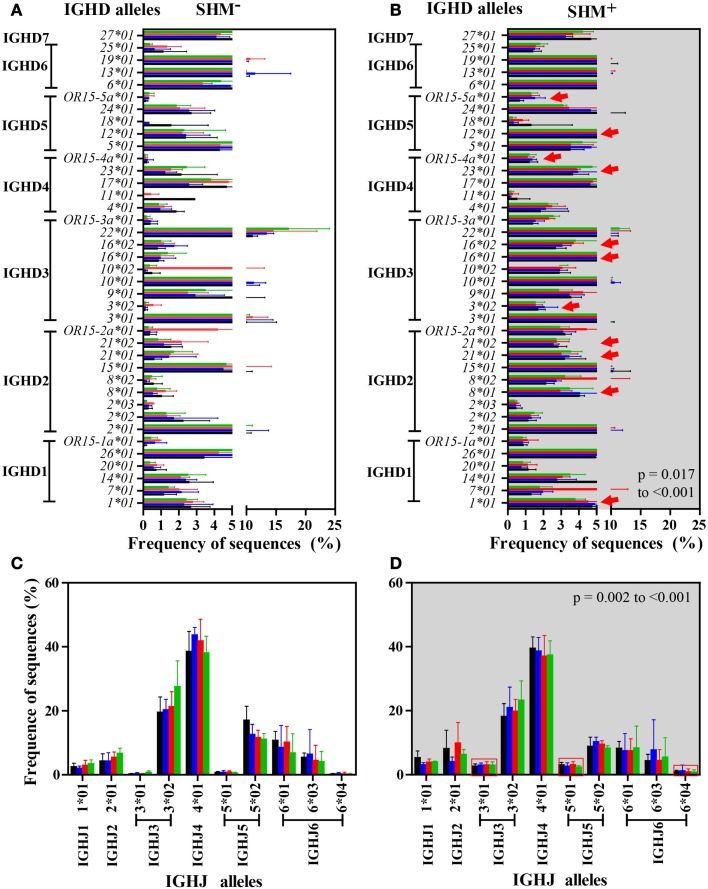
**IGHM IGHD and IGHJ alleles were expressed similarly among study groups but differently between SHM^−^ and SHM^+^ IgM B cells**. Number and frequency of IGHD and IGHJ alleles were similar among the four study groups in IGHM CDRH3 sequences without [IGHD, **(A)**; IGHJ, **(C)**] or with SHM [IGHD, **(B)**; IGHJ, **(D)**]. A significant difference in usage of 11 IGHD alleles, including IGHD1-1*01, IGHD2-8*01, IGHD2-21*01, IGHD2-21*02, IGHD3-3*02, IGHD3-16*01, IGHD3-16*02, IGHD4-23*01, IGHD4/OR15-4a*01, IGHD5-12*01, and IGHD5/OR15-5a*01 (red arrows), and 3 IGHJ alleles, including IGHJ3*01, IGHJ5*01, and IGHJ6*04 (squared by red), was observed across the groups in SHM^+^ sequences in comparison to SHM^−^ sequences (*p* < 0.001 respectively). Bars (mean/SD): HC, black; SLE, blue; HIV-infected individuals: untreated, red; treated, green.

Nearly 90% of the 44 IGHD alleles were detected across the study groups (Figures 7A,B). Sequences with SHM showed significant increases in use of multiple IGHD alleles compared with sequences lacking SHM. Similarly, over 75% of 13 IGHJ alleles, predominantly in IGHJ3 and IGHJ4 families, were identified independent of SHM (Figures 7C,D). A significant increase in three IGHJ alleles was detected in sequences with SHM (Figure 7D). While all study groups had similar IGHD and IGHJ allele usage, the major difference in allele usage was between sequences with or without SHM.

### Biodiversity of allele combinations

**Figure 8:**
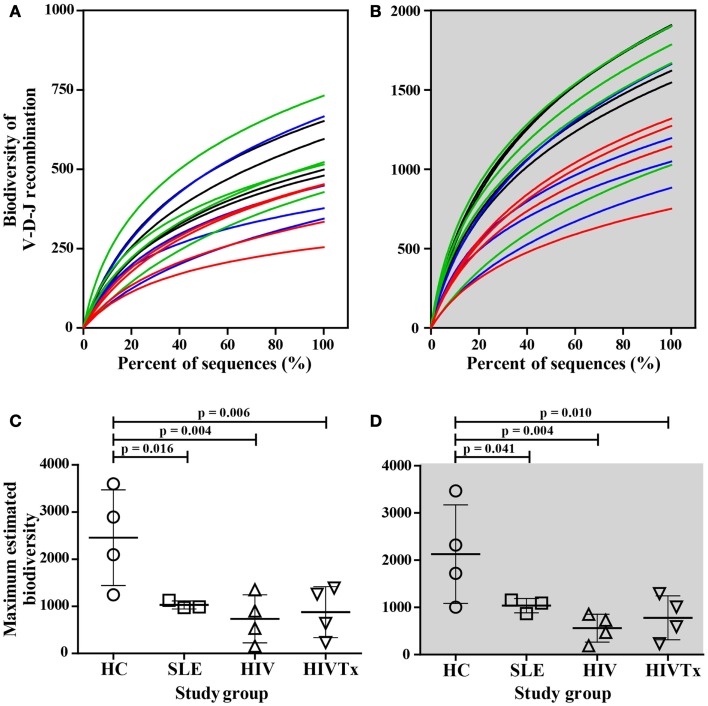
**Significant decrease in biodiversity resulting from V-D-J allele recombination in young adults with SLE and HIV-1 infection in comparison to healthy controls**. As assessed by rarefaction, biodiversity of V-D-J allele recombination along sequence depths decreased in young adults with SLE (blue lines) and untreated HIV-infected individuals (red lines) in comparison to their healthy counterparts (black lines) in IgM B cells without **(A)** or with SHM **(B)**. Control of viral replication by ART failed to restore biodiversity in either cell type [green lines in **(A,B)**]. The maximum biodiversity of V-D-J allele recombination estimated by Chao1 was significantly greater in HC subjects than in individuals with SLE and HIV infection in input IgM B cells without **(C)** or with SHM **(D)** (SLE, *p* = 0.016 or 0.041; HIV, *p* = 0.004 for SHM^+^ or SHM^+^; HIVTx, *p* = 0.006 or 0.01, respectively).

Biodiversity resulting from different combinations of alleles comprising the IGHM CDRH3 regions was assessed in health and disease groups. Every sequence was composed of V-D-J gene segments suggesting intact recombination machinery in all individuals independent of health or disease. When comparing numbers of different combinations along the depth of sequencing using rarefaction curves, a left shift was observed in healthy individuals, indicating greater diversification of combinations whether or not sequences contained SHM (Figures 8A,B). The difference between health and disease was more pronounced when maximum biodiversity was inferred by Chao1 algorithm based on both the richness and evenness of different V-D-J combinations (Figures 8C,D). ART failed to restore biodiversity in either group of IgM transcripts. Overall, reduced diversity of V-D-J combinations was a major contributor to the difference in biodiversity within IGHM CDRH3 sequences between groups.

### IgM sequences resembling bn-HIV-Abs

Combinations of alleles within IgM sequences that might be similar to IgG bn-HIV-Abs were evaluated, but undetected among any individuals (Table 3) (23, 26, 27, 58–68). In contrast, combinations of families found in many bn-HIV-Abs were identified among IgM sequences across the four study groups (Table 4). While few, if any, family combinations similar to M66.6/MPER or CD4 binding site antibodies b12 and VRC-PG04 were detected, combinations similar to other bn-HIV-Abs were identified among IgM sequences in a majority of subjects. In general, family combinations were more frequently represented among IgM sequences with SHM, although frequencies greater than 0.2% appeared in sequences with or without SHM. Frequency of family combinations was not a function of the targets in Envelope, as combinations directed toward CD4 binding site, N-linked glycans, V2/V3, or MPER were detected in almost all individuals.

**Table 3:** **Characteristics of IGHG in bn-HIV-Abs**.

Binding target	bn-HIV-Ab	IGHG				
		IGHV	IGHD	IGHJ	SHM (%)	CDRH3 length (aa)
V2, V3	PG16	3-33*05	3-03*01	6*03	20.5	28
	PG9	3-33*05	3-03*01	6*03	16.7	28
	CH01-CH04	3-20*01	3-10*01	2*01	∼14.3	24
V3 loop	447-52D	3-15*07	3-10*01	6*03	NA	20
Glycans	2G12	3-21*	5-12*	3*	31.7	16
CD4 bs	VRC01	1-02*02	3-16*01	1*01	32.0	14
	VRC02	1-02*02	3-16*01	1*01	32.0	14
	VRC03	1-02*02	3-*	1*01	30.0	16
	VRC-PG04, 04b	1-02*02	5-12*01	2*01	30.0	16
	VRC-CH30-34	1-02*02	3-16*01	4*02	∼25.0	15
	b12	1-03*01	3-10*02	6*03	13.0	20
gp41 MPER	2F5	2-05*	3-03*	6*	15.2	22
	4E10	1-69*	3-16*/6-19*	1*01	15.6	18
	M66.6	5-51*01	3-10*01	6*02	9.3	21

*NA, not available*.

**Table 4:** **IGHV, IGHD, and IGHJ combinations similar to known bn-HIV-Ab in IgM repertoire in peripheral blood**.

bn-HIV-Ab	Target	IGHG			Frequency of sequences (%)																														
		
		IGHV	IGHD	IGHJ	Without SHM																With SHM															
					HC				SLE				HIV				HIVTx				HC				SLE				HIV				HIVTx			
					1	2	3	4	1	2	3	4	1	2	3	4	1	2	3	4	1	2	3	4	1	2	3	4	1	2	3	4	1	2	3	4
VRC-CH30-34	CD4 bs	1*	3*	4*																																
2G12	Glycans	3*	5*	3*																																
CH01-CH04	V2, V3	3*	3*	2*																																
4E10	MPER	1*	3*/6*^a^	1*																																
VRC01, 02, 03	CD4 bs	1*	3*	1*																																
2F5	MPER	2*	3*	6*																																
PG16, 9/447-52D	V2, V3/V3 loop	3*	3*	6*																																
VRC-PG04, 04b	CD4 bs	1*	5*	2*																																
b12	CD4 bs	1*	3*	6*																																
M66.6	MPER	5*	3*	6*																																

*^a^ Sequences in 4E10 align to IGHD3 or IGHD6 with a similar number of nucleotides*.

*White or gray = <0.01% of sequences; yellow = 0.01–0.1% of sequences; orange = >0.1–0.5% of sequences; rust = >0.5% of sequences*.

## Discussion

Little is known about how B cell abnormalities in HIV infection or SLE impact IGH repertoire at the molecular level (1–5, 69–72). Applying deep sequencing to IgM transcriptomes in total B cell populations is a strategy to follow development of the IgM repertoire as a novel molecular assessment at multiple points along the B cell differentiation pathway, which is highly sensitive for detecting perturbations within the repertoire in health and disease. Evaluation of CDRH3 regions in IgM focuses assessment on transition of the repertoire from initial V-D-J recombination in the bone marrow, through antigen-induced clonal expansion, SHM, and establishment of IgM memory B cells (73, 74).

A memory of antigen is imprinted irreversibly by SHM in Ig variable region genes, which renders SHM the most accurate marker to distinguished memory from naïve B cells than B cell phenotype defined by surface expression of IgM, IgD, and CD27. While memory CD27^+^ IgM B cells undergo robust SHM in germinal centers, CD27 is not an immutable indicator of B cell memory (74–77). We observed IGHM sequences with SHM in CDRH3, in accordance with observations suggesting an IgM-expressing memory B cell compartment in the marginal zone (52, 54, 78, 79). The high percentage of IGHM sequences with SHM may be due to a larger IgM memory B cell compartment than previously thought, as well as to increased mRNA levels in IgM B cells with SHM (54), It is unlikely that plasma cell sequences contributed to the results as plasma cell frequency in peripheral blood is minimal (80, 81) and our study design included subjects with no recent immunizations and/or acute infections to minimize the proportion of plasmacytoid B cells undergoing clonal expansion that may skew the IgM sequence repertoire (82).

A small but significant difference was detected between the frequency of SHM^+^ IgM B cells among subjects with SLE, but not HIV-1 infection, compared to healthy individuals (5, 83, 84). These cells resemble recirculating marginal zone memory B cells that contain somatic mutations and can create considerable IGH diversity during early childhood in the absence of specific antigenic stimulation (74, 85).

Rarefaction/Chao1 analysis provides the capacity to compare IGHM biodiversity between sequences expressed by naïve/transitional B cells and IgM memory B cells defined by SHM in peripheral blood B cell populations. Within individuals, CDRH3 sequence biodiversity resulting from V-D-J recombination generated in the bone marrow was significantly limited relative to sequence biodiversity following antigen activation and development of SHM, consistent with estimates of the frequency of SHM within the B cell repertoire (54). A critical finding is that biodiversity of the IgM repertoire in the absence or presence of SHM distinguishes between health and disease. Study groups were similar ages, an important aspect of the design, as age profoundly impacts IGH diversity, particularly with respect to the extent of SHM (86). Overall, IGH affinity maturation is more accurately assessed by direct molecular analysis through pyrosequencing of the IgM transcriptome than by phenotypic analysis of B cell subsets.

While effective treatment restores many HIV-induced B cell defects, overall B cell function remains impaired (8, 72, 87–89). Control of HIV replication by cART failed to restore IgM sequence diversity, although longitudinal studies are needed to evaluate directly the effects of cART on the B cell repertoire. Abnormalities of IgM B cell populations in HIV-1 infection may persist as the result of chronic inflammation that continues to impair B cell populations even when viral replication is optimally controlled (90). Furthermore, persistent low-level viral replication in the secondary lymphoid tissues where B cells and CD4 T-cells co-localize may induce lymphoid apoptosis and/or clonal expansion, contracting the IgM repertoire within memory B cells (4, 91).

We observed an overall shift of distribution of CDRH3 lengths toward shorter lengths in sequences with SHM, but no shifts in hydropathy or charge distribution across the CDRH3 lengths, as found in assessments using sorted IgM memory B cells (51, 92). Differences may reflect a distinct developmental program for CD27^+^ IgM marginal zone B cells that develop outside typical T-dependent or T-independent pathways (85). Overall, neither HIV-1 infection nor SLE produces profound alterations in allele usage, CDRH3 length, hydropathy, or charge distribution within the IgM repertoire, indicating that intrinsic mechanisms for generating junctional diversity and SHM are functional.

Decreased biodiversity in IGHM CDRH3 sequences without SHM supports the observation that HIV-1 reduces the breadth of the IgM repertoire early in B cell development (93, 94). In contrast, reduced biodiversity within the SHM^+^ IGHM repertoire of HIV-infected or SLE subjects might reflect polyclonal expansion, reduced B cell precursors, and/or B cell exhaustion. While pyrosequencing provides an unprecedented view of the human IgM B cell repertoire, depth of sampling of peripheral blood B cell populations was insufficient to detect directly expanded B cell clones, which may be expected from polyclonal expansion. In a study of healthy individuals the estimated IGH pool was greater than 1,000,000 different rearrangements so that even with deep sequencing no single sequence would be represented multiple times (39). Polyclonal expansion of limited number of clones would decrease overall diversity without impacting junctional modification, SHM generation, CDRH3 length distribution, or biochemical characteristics of amino acid residues. The healthy subjects enrolled in our study had no recent illnesses or immunizations that might skew their repertoire (86). In contrast, both the SLE and untreated HIV-infected subjects had active disease, yet CDRH3 length diversity and gene usage was not impacted. Decreased B cell numbers in the peripheral blood are restored by control of viral replication but therapy failed to restore sequence biodiversity in either SHM^+^ or SHM^−^ populations (88, 89). This assessment of the IGHM sequence repertoire provides a basis for examining late stage B cell development through pyrosequencing of IGH in IgG and IgA repertoires within the same mRNA used for the IgM transcriptome, which will expand molecular understanding of the biodiversity of the peripheral blood B cell repertoire in health and disease.

Autoantibodies can result from defective early B cell tolerance in the bone marrow or arise as naïve B cells encounter antigen, undergo SHM and affinity maturation (6). Our study identified no skewing with the IGHM repertoire based on assessment of CDRH3 length, charge, and hydropathy within sequences that contained or lacked SHM, which is not surprising as assessment of the B cell repertoire using clonal sequencing of V-D-J recombination in SLE subjects also failed to reveal major abnormalities (6, 95). Overall, results provide a framework for in-depth studies of the molecular mechanisms that lead to lower biodiversity in SLE and HIV-infected individuals.

An intense area of study in the development of an effective HIV-1 vaccine is the capacity of the repertoire to generate bn-HIV-Abs. Most characterized bn-HIV-Abs are IgG isotype with extensive affinity maturation and SHM (58, 61, 62, 66, 67, 95). IgA autoantibodies, including anti-CCR5 or anti-gp41, produced by a subset of the HIV-1-exposed seronegative individuals or long-term non-progressors, contribute to effective prevention of viral entry at major mucosal portals (29, 32, 96–98). Both IgG and IgA are derived from IgM. Our assessment of the IgM repertoire revealed in all subjects low frequency B cells with the same combinations of IGHV, IGHD, and IGHJ gene families found in known IgG bn-HIV-Abs, particularly those which bind to CD4 binding site, gp41 MPER, V2/V3, glycans in C2, C3, V4 and C4, and V3 loop (23, 26, 27, 58, 60–67, 99, 100). Clearly the IgM repertoire across individuals normally includes B cells with the potential to express IgG bn-HIV-Abs, holding promise that these antibodies can be induced as part of an overall HIV vaccine strategy. Biochemical characteristics of IgM antibody sequences provide an assessment of functional potential of IgM antibodies. In our study, significant differences in frequency distribution of CDRH3 lengths, or distribution of charge or hydropathy along CDRH3 lengths in IgM repertoire of SLE or HIV-infected individuals were found in comparisons with HC. Results suggest that IgM antibodies with characteristics of IgG bn-HIV-Ab were not produced at levels sufficient to perturb IgM repertoire in SLE or HIV-infected individuals.

## Conflict of Interest Statement

The authors declare that the research was conducted in the absence of any commercial or financial relationships that could be construed as a potential conflict of interest.
